# Psychological time as information: the case of boredom^†^

**DOI:** 10.3389/fpsyg.2014.00917

**Published:** 2014-08-21

**Authors:** Dan Zakay

**Affiliations:** ^1^School of Psychology, Interdisciplinary CenterHerzliya, Israel; ^2^Department of Psychology, School of Psychological Sciences, Tel-Aviv UniversityTel-Aviv, Israel

**Keywords:** boredom, prospective-timing, psychological-time, attentional-gate, information-processing-load

## Abstract

The flow of time is experienced by humans although the exact nature of time is not well understood. The importance of time in humans’ life is not in dispute and is reflected by several dimensions like duration, which is best representing the naïve meaning of time. Psychological time serves several important functions which are essential for being able to act and survive in a dynamic environment. In the present paper we argue that psychological time in the form of sensing the pace of the flow of time provides important information to the executive system which control and monitor behavior. When information processing load is below an optimal level for a specific Individual a feeling of boredom is raised. Boredom is accompanied by a slowing of the felt pace of the flow of time. Boredom is a unique mental state which is linked with decreasing efficiency in cognitive and perceptual performance and is correlated with low job satisfaction and general well-being. As such, boredom poses a threat to normal functioning. We suggest that the felt slowing in the flow of time is a signal which, similarly to pain, is aimed at alerting the executive system that resources should be recruited in order to cope with the hazardous state.

## PSYCHOLOGICAL TIME

No understanding of human behavior can be complete without referring to the notion of time. Indeed, humans can sense the flow of time, but the exact nature of the mechanism by which this is done remains unclear. What humans (and maybe animals) experience are temporal experiences, which are subjective feelings that corresponds to physical time. Psychological time is a product of the mind more than a reflection of natural chronometric order (Trautmann, 1995). It refers to temporal dimensions such as duration, pace and the order of perceived and internal events. Psychological time provides our information processing system with important information that enables us to represent the environment in our cognitive system and to act accordingly.

### PROSPECTIVE AND RETROSPECTIVE TIMING

The experience of time is termed prospective when it is related to the duration of an ongoing interval and the observer is aware of the need to judge that duration. When an observer is not aware of the need to judge duration until the termination of a target interval, the experience of time is a retrospective one (Block, 1989). It is not clear if the subjective experiences associated with prospective and retrospective duration judgments are similar, but it is clear that the two types of temporal experiences are based on different information processing processes. Robust empirical findings as well as a comprehensive meta-analysis (Block and Zakay, 1997; Zakay and Block, 2004) indicate that different timing processes underlie the two judgment types. Retrospective duration judgments can be accounted for by the contextual change model (Block and Reed, 1978) which suggests that when retrospective timing is needed, people retrieve from memory contextual changes that were encoded during a target interval. Retrospective duration judgment is a function of the amount of retrieved contextual changes. The more contextual changes are retrieved, the longer the duration is judged to be. As a result, when information processing during an interval is complex (i.e., remembering a complex geometrical figure), the interval is judged to be longer in retrospect than a respective interval in which information processing was simple (i.e., remembering a simple geometrical figure like a circle). This is because complex information processing is causing more contextual changes to be encoded than simple information processing.

In contradistinction, prospective duration judgment is a function of the amount of attentional resources allocated for timing. The more resources are allocated for timing the longer prospective duration judgment is (Brown, 1997). The result is a mirror image of retrospective duration judgment. Prospective duration judgments of same time periods are longer when non-temporal information processing during a target interval is simple than when it is complex. The reason is that the more demanding non-temporal information processing is, the more attentional resources are consumed by it, leaving fewer resources for timing (Zakay, 1999).

### ATTENTION AND PROSPECTIVE TIMING

At any given moment, attentional resources are divided between all the concurrent tasks that need to be carried out simultaneously, including timing (Kahneman, 1973; Zakay, 1989). Zakay and Block (1995) introduced the attentional gate model (AGM) which is based on Church and Gibbon’s (Church, 1984) timing model in animals. An attentional gate was added to the animal model. The gate is controlled by the amount of attentional resources allocated for timing and determines the number of pulses emitted by a pacemaker that can pass through the gate in a time unit. The pacemaker emits the pulses continuously at a constant pace. The pulses are accumulated and counted in a cognitive timer (Wearden, 2004). The more attentional resources are allocated for timing, the more pulses are “allowed” to pass through the gate. Thus, prospective duration judgment is a function of the number of accumulated pulses in a given time period (Zakay and Block, 1997). A similar attention-based model, but with a different gating mechanism was introduced by Lejeune (1998). In this model a dynamic switch is controlled by the attentional resources allocated for timing. The switch is opened and closed at a frequency determined by the amount of attentional resources allocated for timing. With more attentional resources, the higher the frequency and the larger the number of pulses that can pass through, and be accumulated in the cognitive counter (For a comparison between the two models see Zakay, 2000).

The attentional gate as well as the dynamic-switch serves as mechanisms for the regulation of attentional resources between concurrent non-temporal tasks and timing. According to both models when non-temporal tasks are simple and non-demanding prospective timing of same clock time intervals will be longer than when non-temporal tasks are complex and demanding. Because of this state of affairs, prospective duration judgment can serve as a measure of non-temporal information processing load (Zakay and Schub, 1998). The reason for this is that at any given moment attentional resources have to be divided between all concurrent tasks that have to be performed, temporal or non-temporal, and because prospective duration judgment is a function of the amount of attentional resources allocated for timing, prospective duration judgment can be used as a sensitive measure of concurrent non-temporal information processing load. When the load is low, more attentional resources can be allocated for prospective timing and duration estimations become longer as compared with conditions with high non-temporal information processing load. This was validated in several studies (see Brown, 2008) as well as in a meta analytic review (Block et al., 2010).

### FUNCTIONS OF PSYCHOLOGICAL TIME

As was already stated, temporal experiences are essential for enabling humans to represent the temporal aspects of both the external and internal environments, thus enabling adaptation and survival. For example, being able to judge the duration of an event is essential for knowing how to deal with similar events in future encounters. However, we argue that temporal experiences provide the cognitive and meta-cognitive systems with important information which enables optimal monitoring of behavior. Monitoring of spoken communication is just one example (Zakay et al., 2014). In the course of a conversation when party A asks party B a question, a temporal expectation regarding the response latency is evoked. This temporal expectation reflects some kind of a norm. When actual response latency is significantly longer or shorter than the temporal expectation, party a suspects that the response is not based on real knowledge and therefore it can’t be trusted. This indicates that the duration of the response latency is being monitored and timed prospectively. The ongoing prospective duration judgment is compared with the temporal expectation, a process which is well illustrated by the AGM.

In this paper we focus on one specific function of psychological time, namely, providing information about the concurrent level of non-temporal information processing load. This function was not yet elaborated in the literature.

### THE NEED FOR INFORMATION AND FOR INFORMATION PROCESSING LOAD

Humans need a certain amount of information in order to maintain a satisfactory level of adaptive behavior (Kuhltham, 1991). Information is a product of variability in stimulation (Garner, 2014). Woodburn (1957) reports experiments on human behavioral effects following prolonged exposure to a monotonous environment. It was found that under such conditions thinking was impaired, childish emotional responses appeared, visual perception was disturbed, hallucinations developed and brain wave patterns were altered. Similar findings were found in sensory and perceptual deprivation experiments (Zubek and Welch, 1963; Zakay and Lobel, 1983; Grassion, 1986). Similar effects are found in real life situation which resemble perceptual deprivation like in the case of snow-blindness which create a ganzfeld (Avant, 1965). It can be concluded that the need for meaningful information is a genuine need of the cognitive system which strives to gain the optimal amount of information (Merhabian, 1977).

### TEMPORAL EXPERIENCES AS INFORMATION

Michon (1972) introduced the idea of considering time as information. He meant that temporal experiences provide information about the succession of events. Like other perceptual dimensions, psychological time provides our information processing system with important information that enables the representation of the environment such that adaptive behavior becomes possible.

We elaborate on this notion and propose that temporal information informs the executive system which control and monitor behavior about the ongoing state of the system’s performance and functioning. It should be noted that information is more than mere stimulation. This is demonstrated by studies of perceptual deprivation (e.g., Grassion, 1986), in which the amount of information is normal but it lacks variability. More research is needed in order to understand the exact nature of temporal information. For example: How one feels the pace of time? Are retrospective and prospective experiences providing the same type of information or different types? Regardless of the need for more research, the importance of temporal information is clear.

Since any type of behavior takes a certain amount of time, by monitoring the actual time a certain behavior endures and by comparing it to temporal norms or expectations, it is possible to monitor the regularity of behavior. We already gave the example about monitoring the adequacy of spoken communication (e.g., Boltz, 2005). Another example is waiting behavior (Zakay et al., 2009). When one is waiting for an event, and the event is delayed in comparison to the expected waiting duration, a temporal experience of slowing of the pace of time accompanied by a general feeling of tension emerges (Osuna, 1985; Loftus et al., 1987). This signals the system that something is wrong.

Here we focus on the fulfillment of the need for information and for information processing. When this need is not satisfied the system is in danger of not being able to perform optimally, as will be illustrated in the next paragraphs. This state is manifested as an emotion of boredom accompanied by a temporal experience felt as the slowing of the pace of the flow of time or boredom, which signals the system that it is currently engaged with a suboptimal level of non-temporal information processing.

### BOREDOM

Boredom is defined as a unique psychophysiological state possessing interrelated and inseparable emotional, motivational, perceptual, and cognitive concomitants (O’Hanlon, 1981).

Boredom is a common emotion, which can appear as a result of a specific situation or as a typical characteristic of an individual. In the last case we speak about boredom proneness (BP), which is a predisposition to experience boredom (Farmer and Sundberg, 1986).

Boredom is an important issue in psychology, education and work-life, and yet, it is not receiving the adequate attention from researchers, as it deserves. The importance of boredom stems from its links with well-being, psychopathologies, job-satisfaction and other important aspects of human behavior (Smith, 1981).

Situational boredom is experienced when one finds him/herself in a situation in which most of one’s attentional resources are free and are not allocated to a specific task which demands information processing. This might be the result of a monotonous environment which lacks stimulation and variance, or from having to perform a routine, non-challenging task or having to listen to a redundant lecture which does not provide any new information and is read in a monotonous voice. The difference between boredom and situations like leisure or play is that whereas in the latter one is absorbed in the activity and a sense of time disappears, in boredom one wishes to quit the situation and the sense of time is augmented (Csikszentmihalyi, 1990). People characterized as having high BP tend to experience boredom even in situations in which the level of stimulation and required information processing load are normal (Csikszentmihalyi, 2000).

Boredom is maintained by an environment that is perceived as static, with the actor remaining largely disconnected from the processes that comprise the environment (Farmer and Sundberg, 1986). Boredom can be induced experimentally by exposing participants to sensory or perceptual deprivation conditions for long periods (Zakay and Lobel, 1983; Grassion, 1986). In reality, boredom and monotony at work were found to be associated (Drory, 1982).

Boredom and BP are negatively correlated with need for cognition, which indicates a lower level of cognitive motivation than that of other people (Cacioppo et al., 1996). Watt and Blanchard (1994) found that individuals, who were less likely to engage in an enjoyable effortful cognitive activity, were more prone to experience negative affects of boredom when compared to high need-for-cognition persons.

### BOREDOM AND MALADAPTIVE BEHAVIOR

Boredom and BP were found to be linked with maladaptive behavior in several domains.

Whereas boredom was not found to be significantly related to levels of intelligence and education (Hill, 1975), it is recognized as a widespread and significant problem. Boredom and lack of curiosity were reported to be the most common cause of drug use (Samuels and Samuels, 1974), and has been associated with eating disorders for both obese and non-obese persons (Abramson and Stinson, 1977).

Bored students were rated more often as maladjusted by teachers in comparison to other students (Fogelman, 1976). In work-life, job dissatisfaction and diminished performance efficiency, tend to be highly correlated with boredom and BP (O’Hanlon, 1981). BP was found to be a predictor of aggressive and risky driving (Dahlen et al., 2005).

Evidences of an inverse relationship between the ability to cope adaptively with boredom and psychopathology were reported by Hamilton et al. (1984). High boredom- copers reported better well-being and greater compliance with organizational safety rules, compared with low boredom- copers (Annilee, 2007).

Positive correlations between boredom and BP and between level of hopelessness in a hopelessness scale and negative correlations with personal life satisfaction across many dimensions were reported (Neugarten et al., 1961).

### BOREDOM, BP, AND PSYCHOLOGICAL TIME

Based on the former review of prospective timing and attentional model like the AGM, the state of boredom can be defined as a mental state characterized by low level of non-temporal information processing load. The negative emotion which accompanies boredom leads one to wish for the ending of the situation, and therefore, like in waiting, most of a person’s available attentional resources are allocated for prospective timing. The result is a feeling of duration lengthening or slowing of the pace of the flow of time (Zakay, 2012). Note, that even if the feeling of the flow of time might be considered a perceptual illusion (Gruber and Block, 2013; Block and Gruber, 2014), it is still a source of information. This is similar to perceptual illusions like apparent motion or the phi phenomenon which provide significant information, albeit an illusionary one. Not much research has tested the relation between perceived duration and boredom. In one of the BP scales (Farmer and Sundberg, 1986) three items out of 28 relate to time. Watt (1991) found that highly boredom- prone individuals perceived time as passing more slowly during a boring task than low boredom-prone persons, but the two groups did not differ in their objective or chronometric time-passage estimates. Similarly, Wittman and Paulus (2008) report that high boredom-prone individuals perceive a slowing of the pace of time and overestimated durations in time-estimation tasks, when a reproduction method was used. (Note that if a production method is used durations will be underestimated, respectively).

It is of interest to note that the other pole of boredom, termed “flow,” is a state of peak enjoyment, energetic focus and creative concentration experienced by people engaged in adult play which has become the basis of a highly creative approach to living (Csikszentmihalyi, 1990, 2000). From Psychological time perspective, whereas boredom produce a significant increase in duration judgment, flow is a state in which attentional resources are almost fully allocated for non-temporal information processing and as a result duration judgment is minimized (Time flies by when you are having fun; see Zakay, 2012. For more interpretations of this phenomenon see Gable and Poole, 2012, or Sackett et al., 2010).

## CONCLUSION

Psychological time fulfills several vital functions like in the planning and performing of psychomotor activities and movements (Flanagan and Wing, 1997) and in meta-cognition, like in human monitoring of human communication (Zakay et al., 2014). In the present paper we elaborate the notion that psychological time should be considered as information. We further dwell into this consideration and suggest that temporal information is essential in alerting the executive system which control and monitor behavior that the overall level of information processing load is lower than the optimal level required for an adequate functioning of the system. This is manifested by a unique state and emotion called boredom. We reviewed studies showing that boredom and BP are linked with lowering cognitive and perceptual performance, with the use of drugs, with lowering job satisfaction and educational achievements, in reducing the amount of effort one can invest in performing tasks and in low level of need for cognition, and with lowering the level of general well-being. The practical consequences of boredom include diminished performance efficiency and health (O’Hanlon, 1981; Annilee, 2007). This is obviously a hazardous state that is not desired from an evolutionary perspective. People usually wish to find themselves in a mental state of “Flow,” which is the opposite pole of boredom, defined as a state of peak enjoyment, energetic focus, and creative concentration (Csikszentmihalyi, 2000). Indeed, Wilson et al. (2014) found that people preferred to administer electric shock to themselves instead of doing nothing.

In a state of boredom the felt pace of the flow of time is slowed down. This can be explained by attentional models of prospective timing like the AGM (Zakay and Block, 1995). Prospective duration judgment is sensitive to the division of attentional resources between concurrent temporal and non-temporal tasks because it is a function of amount of attentional resources left for timing after the required amount was allocated for concurrent non-temporal tasks. From this perspective, we suggest that the alerting function of psychological time is similar to that of pain. Pain is an unpleasant sensory and emotional experience associated with actual or potential tissue damage. Ecclest and Crombey (1999), suggest that pain is salient in naturally complex environments because the selection of pain interrupts attention, rupture behavior and imposes priority on escape actions. The interruptive function of pain is that pain is selected for action from within complex affective and motivational environments to urge escape. In both cases of boredom and pain, the system is required to change the division of attentional resources in order to cope with the situation.

The alerting function of psychological time is based on a comparison between ongoing prospective temporal judgments and temporal norms and expectations. We suggest that each individual is characterized by an idiosyncratic level of information processing (Merhabian, 1977), required for optimal behavior. When this level is achieved, it is accompanied by a certain felt pace of time. The ongoing felt pace of time is continuously compared with the norm and a state of a too slow pace gives rise to a feeling of boredom.

Further research is needed in order to validate the function of psychological time which was introduced here. A better understanding of the link between boredom, temporal experiences, and information processing might lead to a more complete comprehension of the functions of psychological time on the one hand, and to contribute to the development of effective methods for dealing with the state of boredom and with boredom-proneness.

## Conflict of Interest Statement

The author declares that the research was conducted in the absence of any commercial or financial relationships that could be construed as a potential conflict of interest.
